# Collagen triple helix repeat containing 1 is overexpressed in hepatocellular carcinoma and promotes cell proliferation and motility

**DOI:** 10.3892/ijo.2014.2445

**Published:** 2014-05-19

**Authors:** MASAHIKO TAMEDA, KAZUSHI SUGIMOTO, KATSUYA SHIRAKI, NORIHIKO YAMAMOTO, RYUJI OKAMOTO, MASANOBU USUI, MASAAKI ITO, YOSHIYUKI TAKEI, TSUTOMU NOBORI, TAKAHIRO KOJIMA, HIDEAKI SUZUKI, MASAKO UCHIDA, KAZUHIKO UCHIDA

**Affiliations:** 1 First Department of Internal Medicine, Mie University School of Medicine, Tsu 514-8507, Japan; 2 Department of Molecular and Laboratory Medicine, Mie University School of Medicine, Tsu 514-8507, Japan; 3 Department of Hepatobiliary Pancreatic and Transplant Surgery, Mie University School of Medicine, Tsu 514-8507, Japan; 4 Department of Gastroenterology and Hepatology, Mie University School of Medicine, Tsu 514-8507, Japan; 5 Department of Molecular Biological Oncology, Faculty of Medicine, University of Tsukuba, Tsukuba 305-8575, Japan; 6 Research Division, MCBI, Tsukuba 305-0035, Japan

**Keywords:** hepatocellular carcinoma, collagen triple helix repeat containing 1, comparative genomic hybridization, copy number alteration, integrin β

## Abstract

Although several therapeutic options are available for hepatocellular carcinoma (HCC), the outcome is still very poor. One reason is the complexity of signal transduction in the pathogenesis of HCC. The aim of this study was to identify new HCC-related genes and to investigate the functions of these genes in the pathogenesis and progression of HCC. Whole genomes of 15 surgically resected HCC specimens were examined for copy number alterations with comparative genomic hybridization. Gene expression was compared between HCC and normal liver tissues. The roles of the new genes in the progression of HCC were studied using cultured cell lines. Copy number gain in chromosome 8q was detected in 53% of HCC tissues examined. The gene that coded for collagen triple helix repeat containing 1 (*CTHRC1*), located at chromosome 8q22.3, was overexpressed in HCC compared with normal or liver cirrhosis tissues and identified as a new HCC-related gene. CTHRC1 deletion with short hairpin RNA significantly reduced proliferation, migration and invasion of HepG2 and Huh7 cells. In addition, mRNA of integrins β-2 and β-3 was downregulated, with deletion of CTHRC1 in these cells. Immunohistochemical staining on resected HCC tissues showing positive staining areas for CTHRC1 was significantly greater in poorly-differentiated HCC compared with well-differentiated HCC. Moreover, some cases showed strong staining for CTHRC1 in invasive areas of HCC. *CTHRC1* has the potential to be a new biomarker for the aggressive HCC, and to be a new therapeutic target in treating HCC.

## Introduction

Hepatocellular carcinoma (HCC) is the most common primary malignancy of the liver in adults. It is the 5th most frequent cancer worldwide and the 3rd leading cause of cancer mortality (1). Several approaches have been attempted for the treatment of HCC, such as surgical resection, transarterial chemoembolization, radiofrequency ablation and orthotopic liver transplantation. More recently, an oral multikinase inhibitor, sorafenib, has become a key drug for non-resectable HCC (2). Sorafenib inhibits the serine/threonine kinase activity of Raf-1 and B-Raf, the receptor tyrosine kinase activity of vascular endothelial growth factor receptors (VEGFRs) 1, 2 and 3, and platelet-derived growth factor receptor β (PDGFR-β), the cellular signalings of which are implicated in the molecular pathogenesis of HCC.

Despite the variety of therapeutic options, and the many points of action of sorafenib, the prognosis of HCC is still very poor. Several factors account for the limited efficacy of these treatments. First, most patients have underlying liver disease [e.g., liver cirrhosis due to chronic hepatitis C virus (HCV) or hepatitis B virus (HBV) infection]. Second, HCC has a high rate of recurrence that is caused by intrahepatic metastasis or multicentric occurrence. Another reason for its poor outcome is that HCC is a complex and heterogeneous tumor, and many key signal transduction pathways and molecules other than those targeted by sorafenib are possibly implicated in the pathogenesis of HCC. In addition, many genetic and epigenetic alterations such as copy number alteration (CNA), DNA methylation, or DNA mutation have been suggested as being related to the development of HCC (3,4). Therefore, it is important to have a clear landscape of the genomic aberrations in order to understand the multistep process of HCC progression.

Among the genetic alterations, CNAs can be found in almost all human malignancies. Several attempts have been made to identify CNAs by searching for new genes that are causative for HCC carcinogenesis. In fact, frequent copy number gains at chromosomes 1q, 8q and 20q, and frequent copy number losses at 1p, 4q, 8p, 16q and 17q have been identified in HCC using array-comparative genomic hybridization (CGH) (5–7). However, the roles of these CNAs in the pathogenesis of HCC have yet to be elucidated.

Given these facts, the purpose of the current study was to identify new HCC-related genes by investigating CNAs in the whole genome using array-CGH, and by focusing on specific genes included in the CNA region. Furthermore, by performing functional assays of the identified genes, we aimed to elucidate the role of the genes in the pathogenesis and progression of HCC, and to clarify whether the genes have the potential to be new therapeutic targets.

## Materials and methods

### Patients

Paired tumor and surrounding non-tumor liver tissues (liver cirrhosis) were collected from patients who underwent radical surgery for HCC at Mie University Hospital (Tsu, Japan). Normal liver tissues were obtained from trimmed scrap portions of donor livers used for living donor liver transplantation. In total, liver tissues were obtained from 15 patients and 15 normal subjects. DNA copy number alterations (CNAs) were analyzed in HCC tissues, and gene expression was compared between HCC and normal tissues. Table I shows the patient characteristics. This study was approved by the Institutional Review Board of Mie University Hospital (authorization no. 285). Written informed consent was obtained from each patient included in the study. The study protocol conforms to the ethical guidelines of the 1975 Declaration of Helsinki as reflected in *a priori* approval by the institution’s human research committee.

### Copy number analysis

GeneChip 50K single nucleotide polymorphism (SNP) mapping array analysis was performed according to the standard Single Primer GeneChip Mapping Assay protocol using a Human Mapping 50K Array Hind III (Affymetrix, Santa Clara, CA, USA). Individual SNP copy numbers and chromosomal regions with gains or deletions were evaluated with CNAG 2.0 (8).

### Expression profiling

Oligonucleotide microarray experiments were carried out using Human Genome U133 Plus 2.0 arrays according to the manufacturer’s instructions (Affymetrix). Data were analyzed with GeneSpring GX 7.3.1 (Silicon Genetics, Redwood City, CA, USA).

### HCC cell lines

The human HCC cell lines HepG2 (RCB1648) and Huh7 (RCB1942) were purchased from the Riken Cell Bank (Tsukuba, Japan), Hep3B (ATCC HTB-52) and SK-Hep1 (ATCC HB-8064) were purchased from the American Type Culture Collection (Manassas, VA), and HLE (JCRB0404) and PLC/PRF/5 (JCRB0406) were purchased from the Health Science Research Resources Bank (Osaka, Japan). All cell lines were cultured in Dulbecco’s modified Eagle’s medium (DMEM) (Life Technologies, Tokyo, Japan) supplemented with 1% penicillin/streptomycin (Life Technologies) and 10% fetal calf serum (FCS) (Life Technologies) in a humidified atmosphere containing 5% CO_2_ at 37°C.

### Qualitative reverse transcription polymerase chain reaction (PCR)

The expression of CTHRC1 mRNA in the HCC cell lines was determined by reverse transcription PCR of total RNA. Total RNA was extracted from approximately 10^7^ cells of each cell line with the RNeasy mini kit (Qiagen, Tokyo, Japan), and cDNA was synthesized by extension of oligo dT primers with PrimeScript reverse transcriptase (Takara Bio, Inc., Otsu, Japan). PCR of the cDNA was performed with Ex Taq (Takara Bio). The sense primer used for amplification of CTHRC1 was 5′-AGGGAGGTGGTGGACCTGTAT-3′ and antisense primer was 5′-GCCAACCCAGATAGCAACAT-3′.

### Quantitative real-time PCR

The cDNA of HCC tissues, non-tumorous tissues and HCC cell lines was synthesized from 1 μg of total RNA and quantitative real-time PCR (qRT-PCR) was performed using the ABI prism 7300 Real-time PCR system (Applied Biosystems, Foster City, CA, USA) with EagleTaq master mix kits (Roche Molecular Systems, Branchburg, NJ, USA). The expression levels of target genes from triplicate reactions were determined by normalization to β-actin according to the manufacturer’s instructions. Primer sets are as follows: CTHRC1 forward, 5′-CCAAGGGGAAGCAAAAGG-3′; reverse, 5′-CCCTTGTAAGCACATTCCATTA-3′. Human integrin β-2 forward, 5′-CAGCAATGTGGTCCAACTCA-3′; reverse, 5′-GAGGGCGTTGTGATCCAG-3′. Human integrin β-3 forward, 5′-CGCTAAATTTGAGGAAGAACG-3′; reverse, 5′-GAAGGTAGACGTGGCCTCTTT-3′.

### Western blot analysis

Polyclonal antibody for CTHRC1 was generated by immunization of rabbits. HepG2 cells were fractionated using the ProteoExtract Subcellular Proteome Extraction Kit (Merck Millipore, Darmstadt, Germany) according to the manufacturer’s instructions, and localization of CTHRC1 in HCC cells was determined by western blot analysis. Protein lysates of each fraction were separated by SDS-polyacrylamide gel electrophoresis (12.5%) and transferred to polyvinylidene difluoride membranes. Blots were blocked with 5% milk in Tris-HCl (pH 7.5) with 0.1% Tween-20 for 2 h and proved with primary antibody at 4°C overnight. The immunoblots were then probed with horseradish peroxidase-conjugated anti-rabbit secondary antibody (GE Healthcare, Amersham Place, UK) and visualized using ECL plus (GE Healthcare, Munich, Germany).

### Knockdown of CTHRC1 mRNA

Three types of short hairpin RNA (ShRNA) against CTHRC1 and control ShRNA were constructed using the piGENE vector (Igene, Tokyo, Japan). Their target sequences are listed as follows: Sh1, GAAATGA ATTCAACAATTA; Sh2, AAGGAAGCCCTGAAATGAA; Sh3, AGGGAAAGCTTTGAGGAGT; and control (T7STOP), CACCTTTTTTTT. These ShRNAs and control plasmid were transfected into HepG2 cells and Huh7 cells with FuGENE HD (Roche, Mannheim, Germany), followed by the addition of 1 μg/ml of puromycin after 24 h for selecting transfected cells. Cells were harvested 72 h later for analysis of gene expression, cell proliferation, migration and invasion.

### Cell proliferation assay

Cell proliferation was assessed with the xCELLigence system (Roche Inc., Basel, Switzerland) according to the manufacturer’s instructions. Briefly, each well of a 16-well microtiter plate (E-Plate 16) was filled with 100 μl of DMEM to equilibrate the well membrane, and each plate was incubated for 30 min at 37°C in 5% CO_2_. HCC cells transfected with ShRNA against CTHRC1 or control plasmid were suspended in 100 μl of DMEM and seeded at a density of 1×10^4^ cells per well. Cells were cultured for 48 h with the Real-Time Cell Analyzer (RTCA) single plate (SP) instrument placed in a standard incubator at 37°C in 5% CO_2_. Then, cell proliferation was monitored by recording cell index (CI) values at 15-min intervals for 48 h.

### Cell migration and invasion assays

Cell migratory and invasive abilities were also assessed by the xCELLigence system. Briefly, cell migration was assessed by seeding HCC cells at 2×10^4^/well on a fibronectin-coated CIM-16 plate, and cell invasion was assessed by seeding HCC cells at 3×10^4^/well on a CIM-16 plate coated with Matrigel (Becton-Dickinson, Tokyo, Japan). Both cell invasion and migration were monitored by recording the CI at 15-min intervals for 48 h.

### Real-time PCR array

The changes in gene expression related to cell migration and invasion by CTHRC1 knockdown in HCC cells were analyzed with the Human Extracellular Matrix and Adhesion Molecules RT2 Profiler PCR Array (SABiosciences, Frederick, MD, USA) according to the manufacturer’s instructions. These changes were confirmed also by qRT-PCR.

### Immunohistochemistry

Immunohistochemical staining for CTHRC1 was performed on surgically resected HCC tissues using the Vectastain ABC kit (Vector Laboratories, Burlingame, CA, USA). Deparaffinized sections were heated for 5 min in citrate buffer at 100°C with a pressure cooker to reactivate the antigen, and treated with 0.3% H_2_O_2_ in methanol for 30 min to abolish endogenous peroxidase activity. Sections were blocked with 1% goat serum in phosphate-buffered saline, covered with primary antibody at 4°C overnight, and then covered with second-step biotinylated antibody for 30 min, and incubated with peroxidase-labeled streptavidin for 30 min. After washing, sections were incubated with 0.05% diaminobenzidine/0.15% H_2_O_2_ and counterstained with 10% hematoxylin (Wako Pure Chemical Industries, Ltd., Osaka, Japan).

### Statistical analysis

Comparisons of gene expression, cell proliferation, migration and invasion were performed using the two-tailed Student’s t-test. For differences between rates, Fisher’s exact test was used. p<0.05 was considered statistically significant.

## Results

### CTHRC1 was identified as a new HCC-related gene

**Figure 1:**
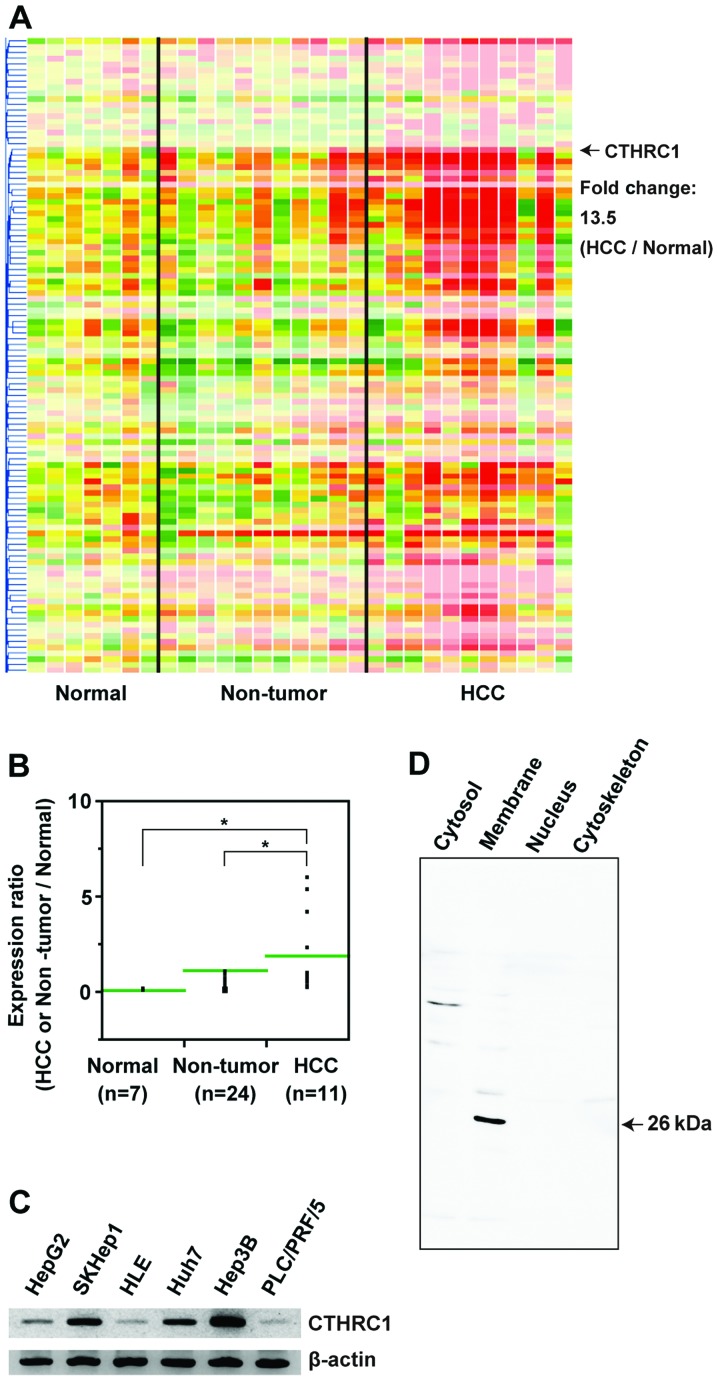
Results of expression analyses. (A) Gene expression profiling of chromosome 8q. *CTHRC1* located at 8q22.3 of which expression level was 13.5-fold higher in HCC compared with normal liver tissues was identified as a new HCC-related gene. (B) Expression levels of CTHRC1 mRNA in each tissue. The expression levels of CTHRC1 mRNA increased from normal tissues to liver cirrhosis and HCC in a stepwise manner (^*^p<0.05). (C) Expression of CTHRC1 mRNA in HCC cell lines. (D) Expression of CTHRC1 in each cell fraction assessed by western blot analysis. A 26-kDa CTHRC1 protein was detected in membrane fractions.

The chromosomes which had frequent CNAs (≥20%) in 15 HCC tissues are listed in Table II. Among them, we focused on 8q, in which CNAs were detected in 53% of HCC tissues, because CNAs in 8q have been also reported very frequently by several groups (9–11), but have not been studied in detail. Through gene expression profiling of 8q, we identified *CTHRC1* located at 8q22.3 as a new HCC-related gene, of which the expression level was higher in HCC compared with normal tissues by 13.5-fold (Fig. 1A). Moreover, re-validation with qRT-PCR revealed that expression levels of CTHRC1 mRNA increased from normal tissues to liver cirrhosis and HCC in a stepwise manner (Fig. 1B). We investigated the expression of CTHRC1 mRNA in HCC cell lines by RT-PCR. As shown in Fig. 1C, CTHRC1 transcripts were detected in all 6 HCC cell lines. Localization of the CTHRC1 protein in HCC cells was also investigated using HepG2. HepG2 cells were fractionated and each fraction was analyzed for CTHRC1 expression by western blot analysis. Fig. 1D shows that the 26-kDa CTHRC1 protein was expressed in membrane fractions of the HepG2 cells.

### CTHRC1 promotes proliferation of HCC cells

**Figure 2:**
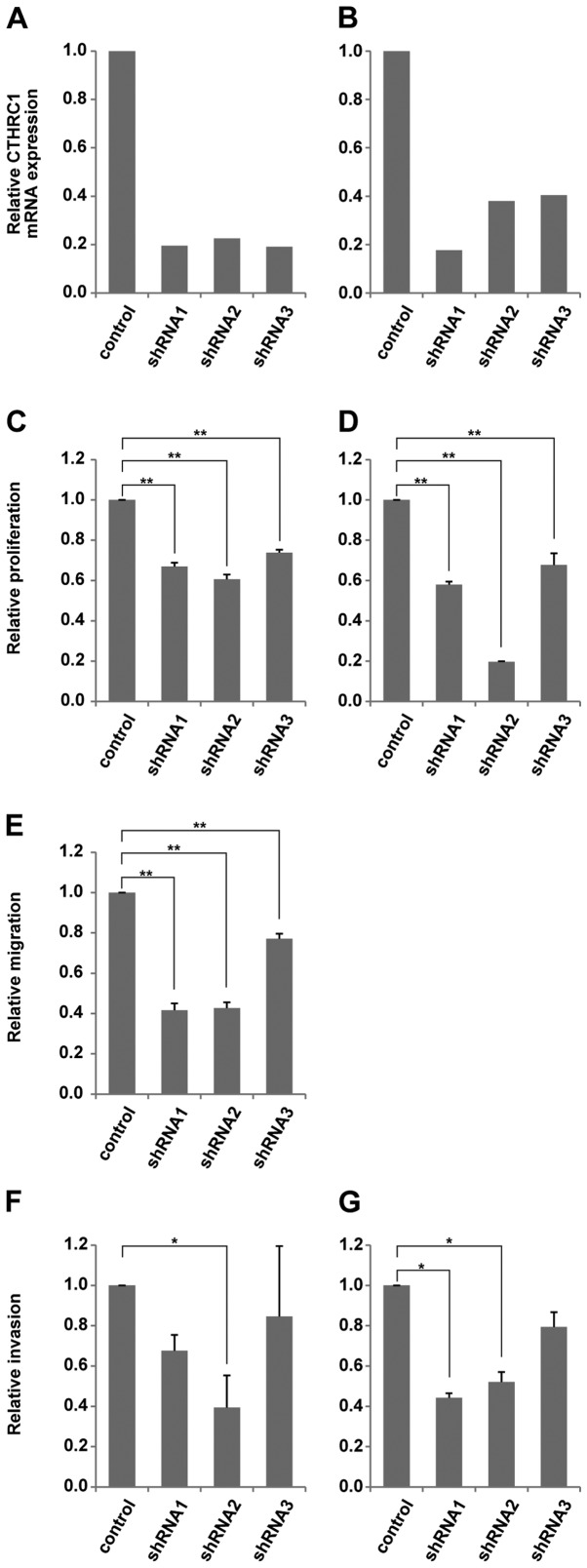
Functional analyses following CTHRC1-deletion in HCC cells. Inhibition of CTHRC1 mRNA in (A) HepG2 and (B) Huh7 with short hairpin (ShRNA). Cell proliferation of (C) HepG2 and (D) Huh7. Cell proliferation after 24 and 48 h was significantly decreased in both the HepG2 and Huh7 cells with CTHRC1 knockdown compared with the control cells. (E) Cell migration of Huh7. Cell invasion of (F) HepG2 and (G) Huh7. Both cell migration and invasion were significantly reduced by CTHRC1 knockdown. The data are expressed as the mean ± SD relative to control of five independent experiments (^*^p<0.05, ^**^p<0.01).

As it has been reported that CTHRC1 is involved in tissue remodeling in rheumatoid arthritis and injured arteries by promoting the migration of fibroblasts (12), we hypothesized that CTHRC1 might have some role in the proliferation and motility of HCC cells. First, we investigated the effect of CTHRC1 on the proliferation of HCC cells. Three types of ShRNA were used to suppress the expression of CTHRC1 in HepG2 and Huh7 cells. The knockdown efficiency was confirmed by qRT-PCR (Fig. 2A and B). As shown in Fig. 2C and D, cell proliferation after 24 and 48 h assessed with xCELLignece system was significantly decreased in both the HepG2 and Huh7 cells with CTHRC1 knockdown compared with the control cells.

### CTHRC1 promotes migration and invasion of HCC cells

We next examined the influence of CTHRC1 knockdown on HCC cell migratory activity. As shown in Fig. 2E, CTHRC1 knockdown caused a significant reduction in cell migration after 24 h in the Huh7 cells compared with the control cells. In addition, the results of the cell invasion assay using the Matrigel-coated plate showed that the cell invasions of both the HepG2 and Huh7 cells after 24 h were also significantly reduced in the CTHRC1-depleted cells compared with the control cells (Fig. 2F and G).

### CTHRC1 knockdown reduces integrin β mRNA in HCC cells

**Figure 3:**
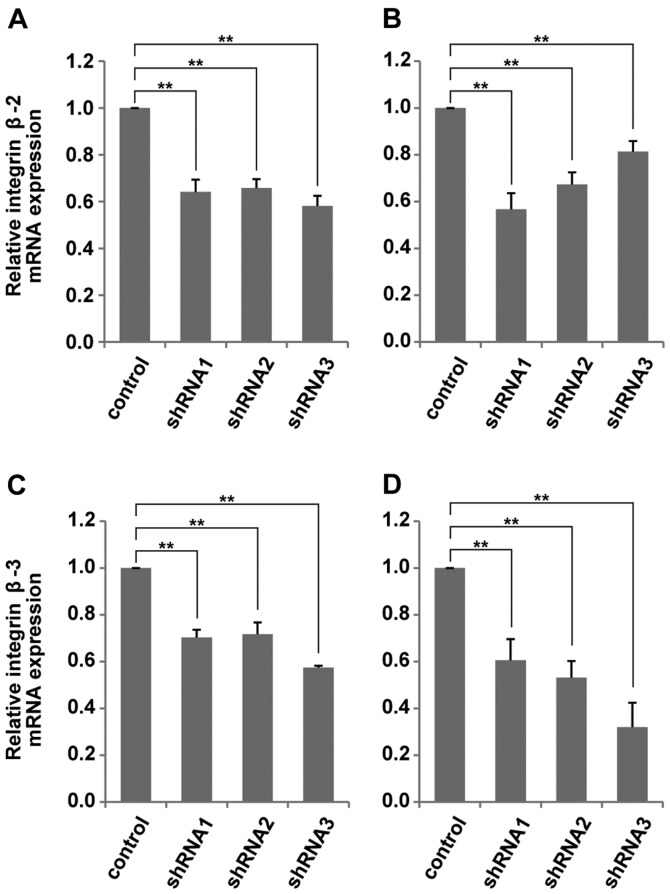
Expression of integrin mRNA following CTHRC1-deletion in HCC cells. (A) Expression of integrin β-2 in HepG2 and (B) Huh7. Expression level of integrin β-2 mRNA was significantly decreased with CTHRC1-depletion in HCC cells. (C) Expression of integrin β-3 in HepG2 and (D) Huh7. Expression level of integrin β-3 mRNA was also significantly decreased with CTHRC1-depletion in HCC cells. The data are expressed as the mean ± SD relative to control of five independent experiments (^**^p<0.01).

To elucidate the mechanism of the suppression of cell migratory and invasive activity in HCC cells by CTHRC1 knockdown, we compared the mRNA expression, which related to cell migration and invasion between the CTHRC1-depleted HepG2 cells and the control cells, using real-time PCR array. The results suggested that the expression of integrin β was affected by suppression of CTHRC1 mRNA (data not shown). To confirm these results, we next performed qRT-PCR to examine the changes in the expression levels of integrin β mRNA in HCC cells after knockdown of CTHRC1. As shown in Fig. 3, mRNA expressions of integrin β-2 and integrin β-3 were significantly reduced with CTHRC1 depletion in both the HepG2 and Huh7 cells. On the other hand, no cell proliferation, migration/invasion, or amount of integrin β mRNA in HCC changed with overexpression of CTHRC1 (data not shown).

### CTHRC1 is expressed in the invasive area of HCC tissues

**Figure 4:**
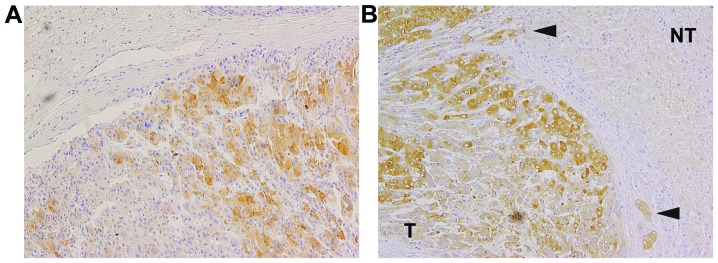
Immunohistochemistry for CTHRC1 on HCC tissues. (A and B) Strong positive staining for CTHRC1 was observed in the HCC cells close to fibrous boundaries, while non-tumor parts were negative for CTHRC1. (B, arrowheads) HCC cells invading surrounding normal liver tissues were strongly positive for CTHRC1. T, tumor; NT, non-tumor. Original magnification, ×200.

Forty-one surgically resected HCC specimens and adjacent non-tumor tissues were examined for CTHRC1 expression by immunohistochemistry. The HCC tissues were divided into three groups according to their degree of differentiation: well-differentiated HCC, moderately-differentiated HCC and poorly-differentiated HCC. The levels of CTHRC1 expression were analyzed in each group and the percentages of positive staining area were divided into three categories. The results are summarized in Table III. The rate for the positive area for CTHRC1 greater than 66% was significantly higher in poorly-differentiated HCC than that of well-differentiated HCC (71.4 vs. 18.2%, p<0.05). The differences of these rates in both between well-differentiated HCC and moderately-differentiated HCC, and between moderately-differentiated HCC and poorly-differentiated HCC were not significant, probably due to the small sample number. None of the patients had positive CTHRC1 staining in the adjacent non-tumorous areas. Interestingly, some patients showed strong positive staining for CTHRC1 in the HCC cells close to fibrous boundaries (Fig. 4) and in invasive areas (Fig. 4B, arrowheads), suggesting high migratory and invasive activity of these cells.

## Discussion

Despite the availability of several therapeutic options, it is still difficult to control the progression of HCC completely. One reason is the complexity of signal transduction in HCC; in other words, a large number of molecules are involved in the pathogenesis of HCC. Therefore, it is important to search for the novel molecules related to HCC progression in order to understand the mechanism of its pathogenesis and to achieve better prognosis. In this study, we identified *CTHRC1* located at chromosome 8q22.3 as a novel HCC-related gene. The results of functional assay showed that CTHRC1-deletion caused reduced cell proliferation and motility in HCC cells. In addition, integrin β mRNA expression was decreased by knockdown of CTHRC1 in HCC cells. Moreover, the CTHRC1 protein was overexpressed in HCC tissues, especially in poorly-differentiated HCCs.

We first analyzed the whole genome for CNA using array-CGH and found copy number gain of 8q in 53% of the HCC tissues. We next performed gene expression analysis of 8q and identified *CTHRC1* as a novel HCC-related gene. Our results showed that CTHRC1 was overexpressed in HCC tissues compared with the surrounding non-tumor tissues. Furthermore, CTHRC1 mRNA was upregulated in all HCC cell lines examined, and the CTHRC1 protein was located in the cell membranes of the HCC cells. The mammalian *CTHRC1* gene was first identified in balloon-injured arteries by Pyagay *et al* (12). This gene is highly conserved among vertebrates. The CTHRC1 protein contains an NH2-terminal peptide for extracellular secretion, a short collagen triple helix repeat of 36 amino acids, and a COOH-terminal globular domain. Although CTHRC1 expression was not detectable in normal arteries, on injury, it was transiently expressed by fibroblasts of the remodeling adventitia and by smooth muscle cells of the neointima. Durmus *et al* (13) reported that CTHRC1 was highly expressed in developing bones and cartilage, the bone matrix and periosteum of adult bones, and in the epithelium-mesenchymal interface including epidermis, airway epithelium, and esophageal epithelium. In addition, these sites of CTHRC1 expression were found to overlap considerably with those reported for the transforming growth factor β (TGF-β) family members and interstitial collagens (13). Kimura *et al* also reported that CTHRC1 increased bone mass as a positive regulator of postnatal bone formation (14). These investigators found that *CTHRC1*-null mice had a decreased number of osteoblasts and low bone mass, while *CTHRC1* transgenic mice had an increased number of osteoblasts and displayed high bone mass. Furthermore, expression of CTHRC1 has been reported in stromal cells of breast cancer (15). Using cDNA arrays, Tang *et al* suggested that the CTHRC1 gene was aberrantly expressed in several types of human solid cancer cells, especially in cancers of the gastrointestinal tract, lung, breast, thyroid, ovarian, cervix, liver and pancreas (16). The results of the present study were congruent with the report by Tang *et al* (16). Although it is still unclear whether CTHRC1 has any function in carcinogenesis, several reports have suggested that inflammation and tissue repair are tightly linked with the development of cancer (17,18). Therefore, it is possible that CTHRC1 plays a certain role in the early stage of carcinogenesis of several cancers, including HCC.

To further elucidate the potential roles of CTHRC1 in HCC progression, we next analyzed the effect of CTHRC1 deletion on the proliferation, migration and invasion of HCC cells. Our results showed that HCC cell proliferation was reduced by the deletion of CTHRC1. Moreover, knockdown of CTHRC1 also reduced the migratory and invasive ability of HCC cells. Several previous studies have suggested that CTHRC1 is related to the motility and invasion of both non-tumor and tumor cells. Using a microarray, Turashvili *et al* found that the *CTHRC1* gene was upregulated in invasive lobular breast carcinoma, suggesting the relation of CTHRC1 to carcinogenesis and cancer progression (19). Using the scratch wound healing assay, Pyagay *et al* showed that both PAC1 cells and embryonic fibroblasts overexpressing CTHRC1 increased migratory activities compared with control cells (12). Tang *et al* reported that CTHRC1 expression was significantly higher in invasive melanoma than in non-invasive melanoma (16). In addition, the experimental results of Tang *et al* with the Boyden chamber also showed that when CTHRC1 expression was inhibited with small interfering RNA (siRNA), migration of melanoma cells was significantly reduced (16). Our experimental results with the real-time cell analyzer indicated that CTHRC1 plays important roles, not only in proliferation or migration, but also in invasion of HCC cells, supporting the findings of previous reports. Therefore, there is a possibility that *CTHRC1* is a new target for therapy of HCC especially for preventing metastasis.

Next, to elucidate the mechanism of how *CTHRC1* affects the motility of HCC cells, we analyzed the changes in mRNA expression. The results of the mRNA array suggested that expression of integrin β-2 and β-3 mRNA was reduced by CTHRC1 deletion; this was confirmed by qRT-PCR. Integrins are a superfamily of transmembrane receptors that form heterodimers of α- and β-subunits. By binding to extracellular matrix (ECM) components, integrins mediate cell adhesion and direct a number of cellular processes such as proliferation, migration and differentiation (20–22). Integrins are also important in promoting cell survival by preventing anoikis, which is apoptosis induced by anchorage-dependent cells detaching from the surrounding ECM (23,24). There has been accumulating evidence showing that integrins β-2 and β-3 have a relationship with cancer progression and migration, including HCC (25–31). For example, Li *et al* reported that the downregulation of integrin β-3 in small cell lung cancer cells reduced cell migration/invasion and induced apoptosis (30). Our results suggest that CTHRC1 promotes the migration/invasion of HCC cells, and also increases cell survival of HCC by inhibiting anoikis via integrin β expression. However, no cell proliferation, migration/invasion, or amount of integrin β mRNA in HCC changed with overexpression of CTHRC1 (data not shown). Presumably, the expression level of CTHRC1 in HCC cells is already sufficient; therefore, increasing its expression does not affect these cell processes to any greater degree. In addition, the detailed mechanism on how CTHRC1 regulates the expression of integrin β remains to be elucidated.

Another possible mechanism by which CTHRC1 mediates cell motility is activation of Wnt signaling. Yamamoto *et al* found that CTHRC1 is a Wnt cofactor protein that selectively activates the planar cell polarity (PCP) pathway by stabilizing ligand-receptor interaction (32). The PCP pathway of Wnt signaling is mediated by the activation of small GTP-binding proteins, including Rac, Rho, Jun N-terminal kinase and Rho-associated kinase, and regulates actin polymerization and cell migration (33,34). Therefore, it is possible that CTHRC1 promotes cell migration through activation of the PCP pathway of Wnt signaling.

Finally, our results of immunohistochemical staining for CTHRC1 showed that poorly-differentiated HCC had a higher expression level of CTHRC1 compared with well-differentiated HCC, supporting previous findings in invasive breast cancer (19). Moreover, strongly positive staining was observed in tumor cells around the cancer borderlines or invasive areas in several cases, suggesting that those cells had a higher activity level of proliferation and migration. These results indicate that *CTHRC1* can be a new biomarker for aggressive HCC.

During preparation of this manuscript, Park *et al* reported the roles of CTHRC1 in pancreas cancer (35) and very recently, Chen *et al* also reported CTHRC1 expression in HCC (36). They found CTHRC1 increased motility and adhesiveness of pancreas cancer cells and HCC cells. Moreover, Chen *et al* also reported HCC with higher CTHRC1 mRNA expression had worse prognosis (36). Our results are clearly supporting these findings. In addition, our data add the information that CTHRC1 protein is actually expressed in human HCC tissues, more prominently in cancer cells which are supposed to be aggressive.

Taken together, the results of the present study obtained from cultured cell lines and human HCC tissues indicated that *CTHRC1* is upregulated in HCC cells and promotes cell proliferation, migration and invasion. *CTHRC1* has the potential to be a new biomarker for types of HCC with poor prognosis, and to be a new therapeutic target for HCC.

## Figures and Tables

**Table I:** Characteristics of HCC patients studied.

Gender	Male	11
	Female	4
Age		62±8.7^a^
HBV serology	Positive	7
	Negative	8
HCV serology	Positive	8
	Negative	7
AFP (ng/ml)		885±3,107^a^
DCP (mAU/ml)		2,168±6,243^a^

a Mean ± SD.

HBV, hepatitis B virus; HCV, hepatitis C virus; AFP, α-fetoprotein; DCP, des-γ-carboxy prothrombin.

**Table II:** Chromosomes which had frequent copy number alterations in HCC.

Gain		Loss	
	
Chromosome	Frequency (%)	Chromosome	Frequency (%)
1q	60	17p	47
8q	53	8p	27
13q	27	16q	27
20q	27	1p	20
6p	27	2q	20
17q	20	13q	20

**Table III:** Summary of immunohistochemical staining.

		Extent of staining area		
		
	Case	0–33%	34–66%	67–100%
Total HCC	41	36.6	24.4	39.0
Well diff	11	63.6	18.2	18.2
Mod diff	23	26.1	34.8	39.1
Poorly diff	7	28.6	0	71.4^a^

Well diff, well-differentiated HCC; mod diff, moderately-differentiated HCC; poorly diff, poorly-differentiated HCC.

a p<0.05 vs. well diff.

## Abbreviations

HCC: hepatocellular carcinoma
CTHRC1: collagen triple helix repeat containing 1
HCV: hepatitis C virus
HBV: hepatitis B virus
CNA: copy number alteration
CGH: comparative genomic hybridization
SNP: single nucleotide polymorphism
qRT-PCR: quantitative real-time polymerase chain reaction
ShRNA: short hairpin RNA
